# Computed tomography in emphysematous pyelonephritis

**DOI:** 10.11604/pamj.2015.22.186.7902

**Published:** 2015-10-23

**Authors:** Erden Erol Ünlüer, Arif Karagöz

**Affiliations:** 1 Izmir Katip Çelebi University Atatürk Training and Research Hospital, Department of Emergency Medicine, Karabaglar, Izmir, Turkey; 2 Izmir Karsiyaka State Hospital, Emergency Department, Karsiyaka, Izmir, Turkey

**Keywords:** Emphysematous pyelonephritis, computed tomography, emergency

## Image in medicine

A 44-year-old woman presented to our emergency department with fever, right flank and abdominal pain for one week. Her past medical history depicted that she had asthma and newly diagnosed diabetes. Direct abdominal X-ray revealed hyperlucent areas in the right kidney lodge (A). Abdominal computed tomography (CT) with contrast showed free gas collection with fatty infiltration outlining the right perirenal space (B). Right kidney also had increased attenuation comparing to the left one. The patient was admitted to the urology department with the diagnose of sepsis due to emphysematous pyelonephritis. Right nephrectomy was recommended to the patient with intravenous antibiotics and drainage. Several classification systems have been proposed to correlate imaging findings with subsequent prognosis and management in patients with emphysematous pyelonephritis. The mildest forms are confined to collecting system or form loculated air-fluid collections in or adjacent kidneys. This may be amenable to percutenous drainage. The more severe infections are characterized by spread of gas into the renal paranchyma and can extend into the perinephric and paranephric spaces and often require emergency nephrectomy as occured in our case. Conventional radiographs may demonstrate a cluster of mottled lucencies within the kidneys.

**Figure 1:**
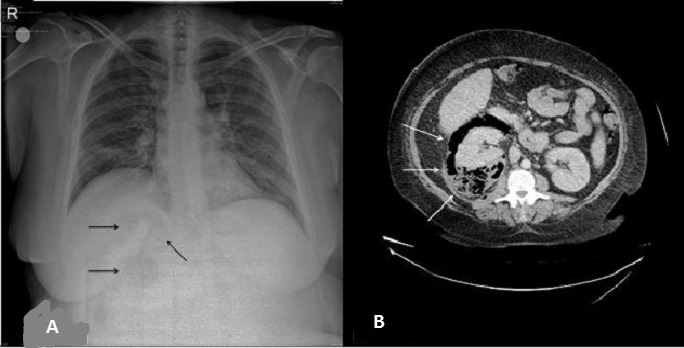
(A) abdominal X-ray and computed tomography showing emphysematous pyelonephritis; (B) ultrasonography shows echogenic foci with ’'dirty’’ shadowing within the kidneys; on CT the collections of gas are seen directly as hypoattenuating foci in the renal lodge

